# Towards molecular selectivity in pharmacoimaging: Comment on van den Bosch and Cools

**DOI:** 10.1162/IMAG.a.1175

**Published:** 2026-03-18

**Authors:** Timothy Lawn, Mitul A. Mehta

**Affiliations:** Athinoula A. Martinos Center for Biomedical Imaging, Department of Radiology, Massachusetts General Hospital and Harvard Medical School, Boston, MA, United States; Department of Neuroimaging, Institute of Psychiatry, Psychology and Neuroscience, King’s College London, London, United Kingdom

**Keywords:** receptor-enriched analysis of functional connectivity by targets, REACT, validation, multimodal imaging, neurotransmitter, pharmacological fMRI

## Abstract

Molecular-enriched fMRI promises to bridge the gap between neurotransmitter systems and macro-scale network dynamics, yet empirical support has remained elusive. Commenting on van den Bosch and Cools (2025), we evaluate the first attempt to validate Receptor-Enriched Analysis of functional Connectivity by Targets (REACT) against individual-level PET data. They found that the effects of methylphenidate on dopamine-enriched networks, but not noradrenaline-enriched networks, tracked individual differences in striatal dopamine synthesis capacity and reward prediction error signaling. While establishing the validity of molecular-enriched networks for this specific use case, the study also exposes critical methodological boundary conditions. We discuss the constraints imposed by spatial collinearity between molecular targets, the influence of state-dependent effects in task-based paradigms, and the necessity of pharmacological blocking studies for establishing causal selectivity. Finally, we look to the future of molecular-informed functional imaging.

The field of neuroimaging has long grappled with a fundamental limitation: the blood-oxygen level dependent (BOLD) signal measured with functional magnetic resonance imaging (fMRI) provides no specificity for the molecular mechanisms that give rise to it. This disconnect between macro-scale network dynamics and micro-scale neurotransmitter function has left pharmaco-fMRI studies abstracted from the molecular targets through which drugs exert their effects (Lawn, Howard, et al., 2023). The recently developed Receptor-Enriched Analysis of functional Connectivity by Targets (REACT) (Dipasquale et al., 2019) method promised to bridge this gap by “enriching” fMRI analyses with spatial information from normative neurotransmitter distributions derived from positron emission tomography (PET, Fig. 1). However, until now, the validity of this approach has remained largely untested.

**Fig. 1.**
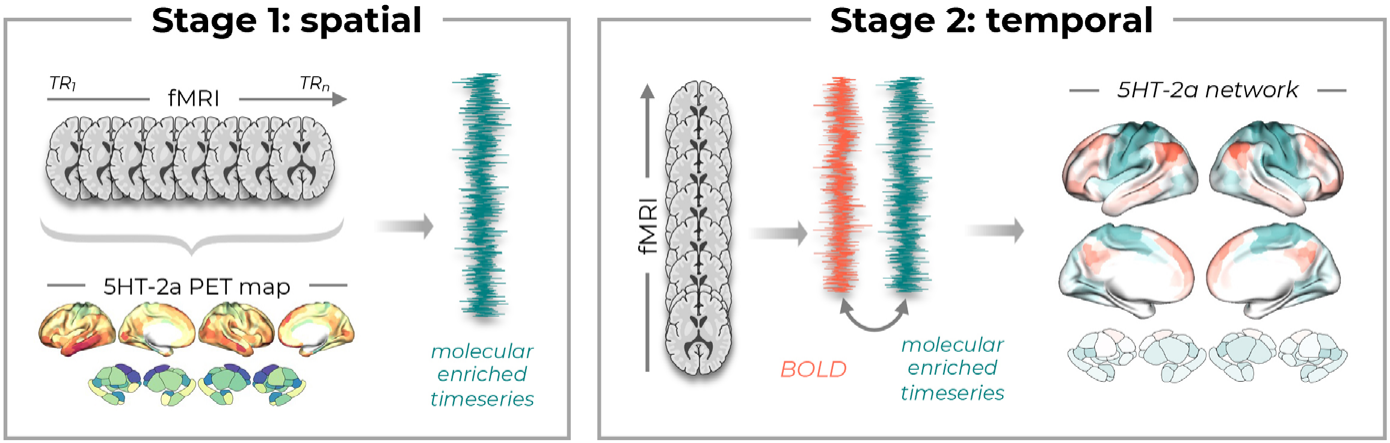
The REACT method. In Stage 1, a spatial regression is performed using a molecular template (e.g., the serotonin 5HT-2a map, bottom) as a spatial predictor against each fMRI time point (TR; top). This process generates a single “molecular timeseries” (teal). In Stage 2 (temporal), this molecular timeseries is correlated with the BOLD timeseries (orange) from each individual region in the brain. The resulting correlation values from all regions create the final “molecular-enriched network” map (right). This captures how each brain region’s activity relates to fluctuations across that molecular system. The stage 1 and stage 2 steps are repeated for each participant, offering subject-level maps that link molecular and systems levels. For more details, see the original paper (Dipasquale et al., 2019) and review (Lawn, Howard, et al., 2023).

In their recent paper, van den Bosch and Cools (2025) provide the first rigorous empirical support for REACT’s molecular selectivity by leveraging a rare dataset combining pharmaco-fMRI with individual-level molecular imaging in the same participants. Their findings represent an important milestone in establishing the credibility of molecular-enriched neuroimaging approaches, offering critical insights into both the method’s potential and pitfalls.

## 1 Testing React’s Molecular Selectivity

The authors used an existing dataset of 85 healthy participants who underwent [^18^F]FDOPA PET scanning to measure dopamine synthesis capacity and pharmaco-fMRI to examine methylphenidate’s effects during a reversal learning task. Methylphenidate acts by blocking both the dopamine (DAT) and noradrenaline (NET) transporters, thereby increasing extra-cellular availability of these neurotransmitters and offering obvious molecular targets to examine using REACT (Hernaus & Mehta, 2016). Previous analysis of this dataset had already established that methylphenidate enhanced neural reward prediction error (RPE) signaling (the canonical functional signature of dopamine) in a manner that varied with individual differences in striatal dopamine synthesis capacity (van den Bosch et al., 2022). Prior work by the REACT developers has also demonstrated that methylphenidate differentially modulates DAT- and NET-enriched networks, with connectivity changes correlating with reinforcement learning behavior (Dipasquale et al., 2020). However, those analyses used normative transporter distributions from population-averaged PET data, without examining whether connectivity changes related to individual differences in actual transporter availability. This left the fundamental question of molecular selectivity unanswered: do these enriched networks truly reflect individual variation in the underlying molecular targets?

Van den Bosch and colleagues answer this question with a compelling “yes,” providing two critical pieces of evidence. First, methylphenidate’s effects on the DAT network, but not the NET network, covaried with individual differences in ventral striatal dopamine synthesis capacity measured by [^18^F]FDOPA PET. Second, the drug’s effects on DAT-enriched connectivity in the left lateral prefrontal cortex correlated with the drug’s effects on neural RPE signaling in approximately the same region. Together, these findings forge powerful links from a drug’s effect on an individual’s neurochemistry, to a specific molecular-related network, and finally to a known functional–behavioral signal.

## 2 Generalizability and the Constraints Imposed by Collinearity

The van den Bosch paper cements discrimination of dopaminergic and noradrenergic mechanisms as a successful use case for REACT. However, that success may not extend to all molecular targets and their varying combinations. Neuromodulatory systems such as dopamine, noradrenaline, and serotonin have widespread projections from subcortical nuclei which may be especially amenable to REACT’s approach of capturing large-scale connectivity patterns. However, neurobiology is not the sole determinant. The authors further used a serotonin transporter (SERT) template as a sensitivity test, reasoning that methylphenidate has relatively weak affinity for SERT compared with DAT and NET. They found substantial spatial overlap between the DAT and SERT templates which resulted in nearly identical drug effects on their molecular-enriched networks. This nuanced problem extends beyond transporters, with a previous study using dopaminergic and serotonergic receptors reporting divergent LSD-induced effects between the dopamine and serotonin systems, but spatial overlap precluded disentangling receptor contributions within each system (Lawn et al., 2022).

This underscores a fundamental constraint of REACT: the method can only discriminate between molecular targets whose spatial distributions are sufficiently distinct at the resolution of MRI. Even if DAT and SERT distributions are distinguishable using higher-resolution techniques such as autoradiography, MRI and PET resolutions remain prohibitive. Thus, while issues of collinearity may not preclude the use of receptor maps for some applications, such as predicting macro-scale functional organization (Hansen et al., 2022), the findings of van den Bosch and Cools demonstrate that it remains a critical barrier for teasing apart the effects of a single pharmacological agent with multiple spatially overlapping targets. As a practical guideline, researchers should examine spatial correlations between molecular templates before applying REACT to discriminate between targets. Based on the DAT/SERT overlap observed here, correlations exceeding *r* > 0.7 likely indicate insufficient spatial distinctiveness for meaningful discrimination at MRI resolution. Variance inflation factors above 5 have also been proposed as a “rule of thumb” for problematic collinearity in REACT models (Lawn, Martins, et al., 2023). These thresholds will, of course, vary as a function of resolution, as voxel or parcel size directly alters levels of collinearity. Future work providing better evidence for when collinearity becomes problematic and strategies to mitigate it will be critical for the meaningful application of REACT.

## 3 State Dependence

A second critical insight from this study stems from its use of task-based rather than resting-state fMRI data. Previous REACT studies have predominantly used resting-state data, but prior work has shown that drug effects on brain activity and connectivity can be highly context dependent (Kowalczyk et al., 2022; Pfeffer et al., 2021; Shine et al., 2018). Indeed, van den Bosch and Cools (2025) found more extensive drug modulatory effects during task performance than had been observed at rest (Dipasquale et al., 2020). This suggests that task demands create synergistic effects between drug action and neural activity, where the combination of pharmacological modulation and task-driven activation produces more pronounced network changes than either factor alone. Thus, state dependence is a critical methodological consideration as network effects observed at rest may not capture the full pharmacological impact of a drug.

## 4 Establishing Causality

While van den Bosch and Cools provide important empirical support through individual-level PET data, the field would benefit from causal manipulations. Salvan et al demonstrated that serotonin-enriched networks are modulated by optogenetic stimulation of the dorsal raphe nucleus, the primary source of cortical serotonin (Salvan et al., 2023). While this was a powerful causal manipulation, the authors did not demonstrate that this effect was unique to serotonin-enriched networks over, for example, a dopamine-enriched network, leaving open the possibility of non-specific BOLD effects. The work by van den Bosch and Cools is, therefore, a critical complement, providing exactly this molecular-level specificity, albeit through correlation with individual differences in PET measures rather than direct stimulation.

The gold standard for achieving both causal and molecular selectivity is the pharmacological blocking study: comparing drug effects alone versus drug effects in the presence of selective antagonists. For example, co-administration of ketanserin (a 5-HT_2A_ antagonist) alongside LSD blocks its subjective and neural effects (Preller et al., 2018). While LSD has multiple molecular targets beyond 5-HT_2A_ (Jain et al., 2025), such existing datasets could validate REACT by demonstrating that serotonin-enriched network effects are selectively abolished under ketanserin co-administration while other network effects remain intact. Ideally, comprehensive validation would extend to multiple selective antagonists to fully characterize a drug’s effects across its primary and secondary targets. For instance, a drug acting on both D2 and 5-HT_2A_ receptors could be tested with D2 blockade (e.g., sulpiride), 5-HT_2A_ blockade (e.g., ketanserin), or both, to determine which receptor mediates which network effects. This approach would provide causal evidence for the molecular selectivity of each enriched network. However, the limited availability of such agents for human use remains a significant translational hurdle and highlights the importance of preclinical animal imaging where causal manipulations are more feasible.

## 5 From Molecules to Networks to Symptoms

The ability to reliably link the pharmacological targets of a drug through to a non-invasive measure of brain function opens exciting possibilities for clinical applications. Rather than relying on complex multi-drug designs to infer molecular mechanisms, REACT could enable simpler pharmaco-fMRI studies to dissect the molecular basis of drug effects, providing value throughout the drug discovery pipeline. REACT has shown promise in characterizing transdiagnostic symptomatology (Lawn, Howard, et al., 2023) and predicting treatment response (Martins et al., 2022; Tolle et al., 2025). This suggests the method may not only bridge the gap from molecular to systems-level dynamics in pharmaco-fMRI, but also between neural (dys)function and pharmacological mechanisms, potentially enabling molecularly specific intervention from non-invasive testing. However, the field must proceed cautiously. This clinical utility hinges on the core assumption that population-averaged molecular templates from healthy individuals are applicable to clinical populations despite potential pathology-induced alterations in receptor density or binding. Although work to date indirectly supports this assumption, it remains a critical vulnerability that requires validation. Future work comparing patient-specific molecular imaging data with normative templates will be crucial for establishing the clinical validity and utility of REACT.

## 6 Looking Forward

**Fig. 2.**
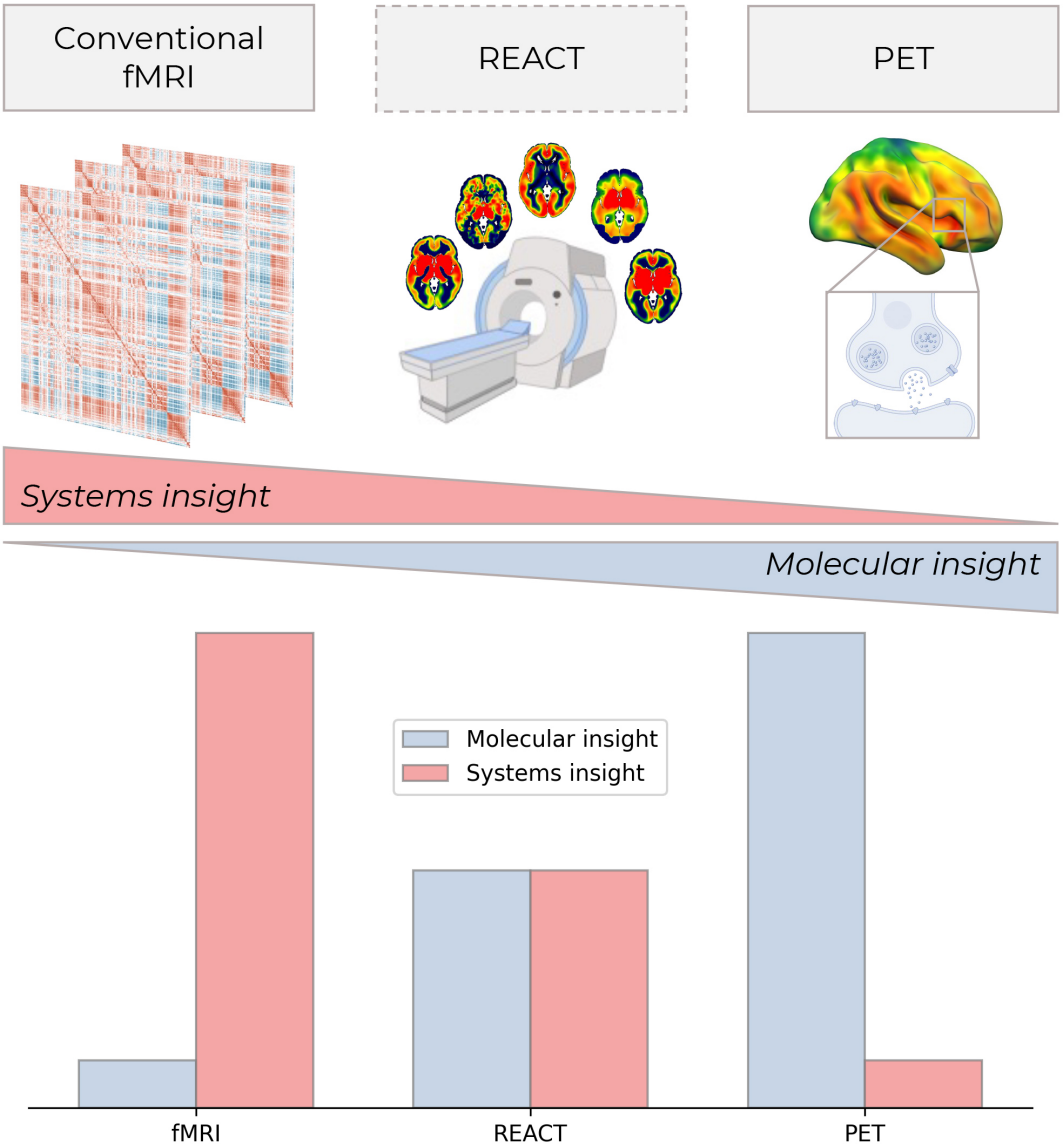
REACT’s methodological position between conventional fMRI and PET. REACT provides a hybrid approach that balances systems-level and molecular insights, offering the specialization of neither domain, but incorporating elements of both to bridge organizational scales.

The promise of molecular-enriched neuroimaging lies in the unique methodological position between conventional fMRI and PET (Fig. 2). Rather than replacing either approach, REACT bridges the explanatory gap between molecular targets of drugs and network-level dysfunction in disease. Van den Bosch and Cools have taken an important step toward realizing this promise by demonstrating that, under appropriate conditions, REACT can indeed partly overcome the lack of molecular selectivity of conventional pharmacoimaging. Delivering upon this promise, however, will depend on several critical next steps. These include expanding this validation across neurotransmitter systems, determining the optimal contexts for applying REACT to different molecular targets, implementing blocking studies to firmly establish causal relationships, and demonstrating the validity of healthy group-average templates within clinical populations. The continued development and open dissemination of high-resolution molecular atlases, derived from large cohorts of PET and SPECT imaging (Beliveau et al., 2017; Hansen et al., 2022), will be foundational to this effort. The use of molecular informed functional imaging may, therefore, be an important component of bringing functional imaging closer to real-world pharmaceutical development and clinical applications.

## Data Availability

No new data were generated or analyzed in this commentary.
